# A health economics assessment of self-care with over-the-counter ibuprofen in dysmenorrhoea, migraine and acute rhinosinusitis in the United Kingdom

**DOI:** 10.1186/s12962-025-00660-6

**Published:** 2025-10-15

**Authors:** Daniela Afonso, Amy Dymond, Isabel Eastwood, William Green, William Laughey, Patricia Aluko, Graham Pennick, Imran Lodhi, Bruce Charlesworth

**Affiliations:** 1 York Health Economics Consortium (YHEC), University of York, Enterprise House, Innovation Way, York, YO10 5NQ UK; 2 Reckitt Healthcare Ltd, Hull, UK; 3 Hull York Medical School, University of York, York, UK

**Keywords:** Selfcare, Ibuprofen, Primary and secondary care, Economic evaluation, Absenteeism

## Abstract

**Background:**

Increased appropriate use of self-care for minor conditions can reduce the number of healthcare professional appointments and, hence, provide opportunity cost savings to the National Health Service (NHS). The receipt of over-the-counter medications can lead to faster access to treatment, improved health-related quality of life, and fewer education and work days lost.

**Methods:**

A model was developed to evaluate the economic impact of a change in the proportion of people using self-care with ibuprofen to manage three conditions (dysmenorrhoea, migraine and acute rhinosinusitis) on preventable healthcare resource use from the perspective of the NHS and Personal Social Services (PSS). The total number of appointments for each condition was estimated from NHS Digital and was based on the number of primary (*n* = 230,298,091) and secondary (*n* = 22,839,832) care visits, and the proportion of visits due to each condition (informed by clinical opinion). Work and school days lost were also modelled to estimate the wider indirect costs associated with preventable absences due to delays in receiving treatment. Deterministic sensitivity and scenario analyses were also conducted to estimate the uncertainty associated with the analysis.

**Results:**

The use of self-care with ibuprofen was increased by 5% in the base case analysis. The results indicate that this increase could prevent 409,243 appointments in the United Kingdom over a one-year time horizon. 882,875 and 117,114 work and school hours lost could also be prevented, respectively. Sensitivity analysis suggests the magnitude of change in self-care, average working hours/pay and appointment waiting times are the main drivers of the model results.

**Conclusion:**

Self-care with ibuprofen provides opportunity cost-savings to the NHS and frees up the capacity of healthcare professionals so that they can focus on more severe conditions.

**Supplementary Information:**

The online version contains supplementary material available at 10.1186/s12962-025-00660-6.

## Introduction

Self-care covers all actions that individuals can take to prevent or care for conditions and includes over-the-counter (OTC) medicines, monitoring devices, diagnostic tools and help support groups [1]. Self-care improves health-related quality of life (HRQoL) by putting people in control of their health and wellbeing, increasing self-confidence and improving health literacy [1]. National Health Service (NHS) England recommends that people with minor conditions (those that do not last long, require little or no medical intervention, and can be treated with OTC medicines purchased from pharmacies or supermarkets) should not be routinely prescribed medication in a healthcare appointment [1, 2]. Inconsistent information on available services, and how they should be accessed, leads people to seek support from accident and emergency (A&E) departments and general practitioners (GPs) when self-treating would be an appropriate option [3]. People who do not use self-care may experience continuation of symptoms unnecessarily due to delays between symptom onset and speaking to a healthcare professional [4].

Those experiencing minor conditions could receive more convenient, faster access to treatment through self-care, leading to reductions in symptom burden, fewer lost work and education days, and greater economic welfare [4]. Additionally, the prevention of unnecessary appointments provides opportunity cost savings to the NHS, allowing professionals to focus on more severe conditions. Evidence suggests that European countries, including the United Kingdom (UK), could make significant healthcare savings by moving certain prescription-only drugs to OTC medicines [5]. Similarly, savings could also be anticipated by implementing strategies that make greater use of OTC medications, where appropriate.

Everyday pains – including headaches, migraines, backaches, and musculoskeletal pains – are common. Men and women experience 1.2 and 1.6 pain occasions per month (excluding female-specific pain such as dysmenorrhoea), respectively [6]. Dysmenorrhoea affects over 70% of women under 25 years of age worldwide [7]. There is also a significant prevalence of aches and feverish symptoms that accompany common viral infections: globally, the mean incidence of the common cold is 2.25 episodes per person per year. Given the frequency and self-limiting severity of these everyday pain occasions, the importance of self-care (including OTC medications) has not yet been widely quantified [8].

A model was developed to estimate the economic implications of a change in the proportion of people using self-care to manage self-limiting conditions. Specifically, a model was developed from an NHS and personal social services (PSS) perspective to evaluate preventable healthcare use via ibuprofen purchased OTC for three self-limiting conditions – dysmenorrhoea, migraine and acute rhinosinusitis (common cold). The model analysis also included out-of-pocket and societal perspectives to understand the wider indirect costs associated with preventable work/school absences due to delays in receiving treatment.

## Methods

### Patient population

The model focused on people seeking a healthcare appointment due to dysmenorrhoea, migraine, or acute rhinosinusitis. These conditions were chosen because their symptoms can typically be treated with OTC medication. Ibuprofen was chosen as the treatment option because it is recommended by the National Institute for Health and Care Excellence (NICE) as the first-line treatment for migraine and dysmenorrhoea and as a self-management strategy for acute rhinosinusitis [9–12].

### Model overview

**Fig. 1:**
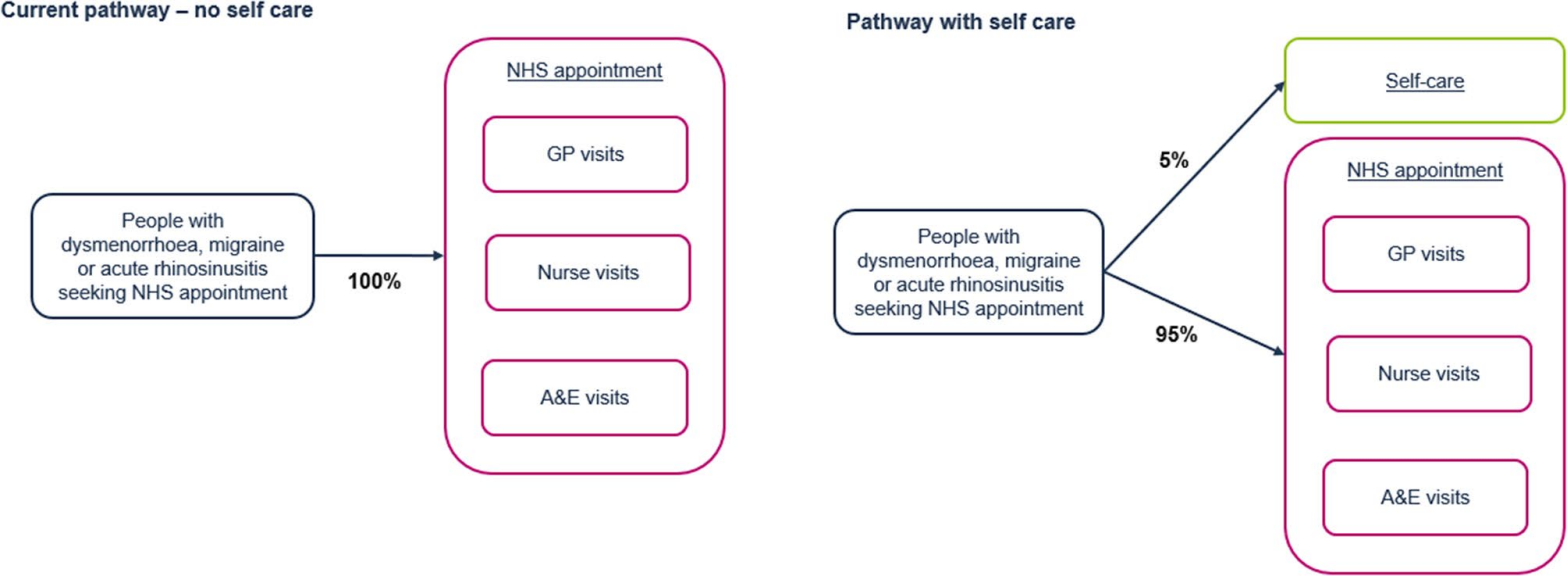
Model structure. A&E, Accident and emergency; GP, General practitioner

A cost calculator, outlined in Fig. 1, was developed in Microsoft Excel to quantify the economic impact following a change in the proportion of people using self-care in terms of preventable healthcare appointments and school/work absences.

The model estimated the total number of annual appointments attended due to dysmenorrhoea (2,315,730), migraine (2,381,061) and acute rhinosinusitis (3,488,071) from the 1st of April 2021 to 31st of March 2022 in English NHS hospitals, as well as NHS-commissioned activity in the independent sector in England [13, 14]. It was assumed that at least a proportion of these appointments could be reduced via the use of OTC treatments, which can be used to relieve the symptoms of each condition (further details below). The appointments were stratified into primary and secondary care. Primary care appointments were further stratified into GP and nurse appointments, and by in-person and telephone appointments. All secondary appointments were modelled as in-person A&E visits.

The model estimated a change in the number of primary and secondary care appointments following an increase or decrease in the uptake of self-care. An increase in self-care was hypothesised to reduce the number of appointments. Hence, the model captured potential opportunity cost savings to the NHS through increased availability of appointments for other reasons. A targeted literature search was conducted to inform the proportion of people who would take time off work for each of the conditions, however, no relevant data were identified. Therefore, it was assumed that 50% of people with a migraine and 25% of people with dysmenorrhoea or acute rhinosinusitis, who do not use self-care, would take time off work and school whilst waiting for an appointment – this accounted for the fact that a proportion of people would continue to attend despite symptoms. People who used self-care were assumed to have their symptoms relieved more quickly and without the need for any healthcare appointments. Therefore, they did not experience the absences from work and school associated with those who waited for an appointment.

Following consultation with two clinical experts (one GP and one A&E consultant), and in alignment with NHS guidance, it was assumed that ibuprofen would not be prescribed for these conditions [2]. Therefore, the whole modelled population, including those who attended healthcare appointments, incurred the cost of purchasing OTC ibuprofen from a supermarket or pharmacy. However, a scenario analysis was run whereby ibuprofen could not be purchased as OTC; instead, those eligible for a free prescription were assumed to receive an ibuprofen prescription, while the remaining people incurred an NHS prescription charge of £9.95 [15].

The impact of a 5% increase in the proportion of people using self-care (i.e. a 5% reduction in the number of primary and secondary care appointments) was modelled in the base case. Discounting was not necessary because the time horizon was below one year. The model estimated the economic impact of a change in self-care over a one-year time horizon from the following three perspectives:

- The NHS and PSS: The opportunity cost of primary and secondary care appointments, with prescription costs considered in a scenario analysis.
- Out-of-pocket: OTC medication costs.
- Societal perspective: Work and school days lost due to symptoms.

### Model inputs

The key model inputs are presented in Table 1. Model inputs were obtained from a targeted literature review.

#### Number of primary and secondary appointments per year

The total number of primary care appointments attended per year in the UK was sourced from published literature [13]. However, these data were not stratified by the reason for each appointment. Therefore, the aforementioned clinical experts informed the proportion of primary care appointments related to dysmenorrhoea, migraine and acute rhinosinusitis [16, 17]. The total number of primary care appointments was multiplied by the relevant proportions to estimate the total number of annual primary care appointments associated with each condition. These were divided into in-person and telephone appointments based on appointment mode data from NHS Digital [13] (Supplementary Material Table S.1).

The number of A&E visits attended annually due to each condition was sourced from NHS Digital Hospital Accident and Emergency Activity 2021/22 [14].

The dysmenorrhoea population was limited to those aged 12 to 51 years old in alignment with clinical advice (Supplementary Material Table S.2).

#### Costs

The following costs were included in the model: healthcare appointments, medication and productivity loss. The frequency of each resource use was multiplied by the unit cost, and costs were aggregated to compare the total costs with current levels of self-care and following a change in the proportion of people using self-care. Costs were sourced from published data and based on the 2022/2023 cost year [18, 19].

The total cost of medication for each indication was calculated based on the average length per episode (3 days for dysmenorrhoea, 1.54 days for migraine and 2 days for acute rhinosinusitis) [20–22]. It was assumed that people received treatment for as long as the symptoms were present. The dose of ibuprofen required for each condition was obtained from the NICE British National Formulary (BNF), with a separate dose applied to those under and over 12 years old [23]. Following clinical consultation, it was assumed that all adults would take ibuprofen in a tablet formulation and 98% of those under 12 years old would use an oral suspension. The inputs used to estimate the total cost of medication are presented in Supplementary Material Table S.3.

In alignment with current NHS policy guidance, the base case analysis assumed that everyone in the model would purchase OTC ibuprofen [2]. Therefore, the cost of ibuprofen was based on a prominent UK pharmacy; these costs were applied to the out-of-pocket perspective (Supplementary Material Table S.3) [24–26].

### Work and school hours lost

The average waiting times associated with primary and secondary care appointments (1.28 days and 3.90 h, respectively [13, 14]) were adjusted to account for the average number of working and school hours per week in the UK. For example, the wait required to receive treatment in A&E would not result in absence if a person working weekdays experienced symptoms on a weekend.

The length of school absence per appointment was multiplied by the proportion of the UK population between the ages of 5 and 17 years (i.e. in full-time education) (Supplementary Material Table S.2). The length and cost of work-related absence were multiplied by the proportion of the population between the ages of 18 and 65 years (assuming all people in this age category were in employment and retired at 65 years) (Supplementary Material Table S.2) [27].

The median average hourly UK wage was multiplied by the number of days missed per appointment and the number of appointments to estimate the total costs associated with absenteeism under current, and a change in, levels of self-care [28]. The cost associated with this productivity loss was applied to the societal perspective (Supplementary Material Table S.3).

**Table 1:** Key model input parameters

Number of visits per year under current practice	Dysmenorrhoea	Migraine	Acute rhinosinusitis	Source
Number of GP appointments: In person	1,122,310	1,122,310	1,683,465	NHS Digital and clinician consultation [13]
Number of GP appointments: Telephone	463,354	463,354	695,031	
Number of nurse appointments: In-person	507,706	507,706	761,559	
Number of nurse appointments: Telephone	209,610	209,610	314,415	
Number of A&E visits per year under current practice	12,749	78,080	33,600	NHS Digital [13, 14] SNOMED CT IDs: 266,599,000, 37,796,009, and 36,971,009.
**Cost and resource use**	**Input**			**Source**
Cost per GP appointment (in person)	£41.00			PSSRU 2022 [18]
Cost per GP appointment (telephone)	£15.50			
Cost per nurse appointment (in person)	£17.06			PSSRU 2022 [18], HEIW [29]
Cost per nurse appointment (telephone)	£8.09			
Cost per A&E attendance	£117			NHS Cost Collection 2021/22 [19]
**Cost and resource use**	**Input**			**Source**
Average waiting time to see a GP or nurse following symptom onset	1.28 days			NHS Digital [13]
Average waiting time following presentation in A&E	3.90 h			NHS Digital [14]

A&E, Accident and emergency; GP, General practitioner; HEIW, Health Education and Improvement Wales; NHS, National Health Service; PSSRU, Personal Social Services Research Unit

### Deterministic sensitivity and scenario analysis

Deterministic sensitivity analysis (DSA) was conducted to investigate first-order uncertainty by varying parameter values, within realistic ranges, to determine the impact on model results. Parameters were varied individually by +/-25% and presented in a tornado diagram, which summarised the impact of changing each parameter on the model results and ranked the size of the individual impact from top to bottom. A series of deterministic scenario analyses were performed to assess the robustness of the results when different inputs were varied. A description of the scenarios performed is provided in the Supplementary Material S.3.

## Results

### Base case analysis

The base case results indicate an increase in ibuprofen uptake by 5% could prevent 409,243 NHS appointments in England when compared with current levels of self-care for the three conditions evaluated in this study (Table 2 and Supplementary Material Table S.4). This equates to opportunity cost savings of £11,852,342 to the NHS. These savings are predominantly due to the reduction in GP and nurse visits (contributing to 79% and 15% of the cost-savings to the NHS and PSS, respectively). Increased self-care uptake is also predicted to substantially reduce the number of education and work hours lost over one year. The increase in self-care uptake does not impact out-of-pocket costs because, in the base case analysis, it was assumed that everyone purchased ibuprofen themselves, regardless of whether they attended an NHS appointment.

**Table 2:** Summary of base case economic results over a one-year time horizon

	Change in self-care	Current pathway	Difference
**Total costs**			
Total NHS and PSS costs	£225,194,499	£237,046,841	-£11,852,342
Total out-of-pocket costs	£16,269,415	£16,269,415	£0
Total societal costs	£163,743,835	£172,361,932	-£8,618,097
Total costs	£405,207,749	£425,678,188	-£20,470,439
**Work and school hours lost**			
Work time lost	16,774,620 h	17,657,495 h	-882,875 h
School time lost	2,225,167 h	2,342,281 h	-117,114 h
Total	18,999,788 h	19,999,777 h	-999,989 h
**Number of appointments**			
GP visits	5,272,335	5,549,826	-277,491
Nurse visits	2,385,077	2,510,607	-125,530
A&E visits	118,208	124,429	-6,221
Total number of visits	7,775,619	8,184,862	-409,243

A&E, Accident and emergency; GP, General practitioner; NHS, National Health Service; PSS, Personal Social

### Sensitivity analysis

**Fig. 2:**
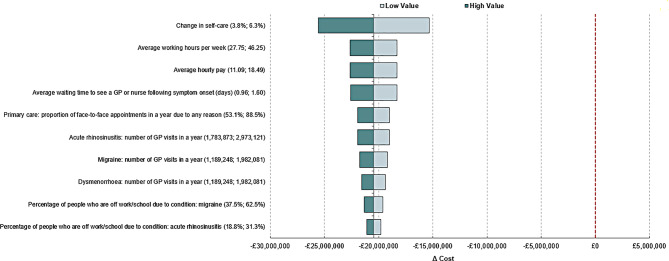
Tornado diagram: All populations and perspectives combined. A&E, Accident and emergency; GP, General practitioner; NHS, National Health Service

The results of the DSA (Fig. 2) show that the percentage change in self-care is the primary driver of the model when all other inputs remain constant. The average working hours per week, the average hourly pay and the average waiting time to see a GP or nurse following symptom onset were also key drivers of the model. No inputs cause the increase in self-care uptake to become cost-incurring. Tornado diagrams for each condition are presented in the Supplementary Material Table S.4.

Additional sensitivity analysis was conducted to investigate the impact on the incremental cost when the uptake of self-care was varied (Supplementary Material Figure S.2). The results show that a reduction in self-care of 50% and 75% could increase potential costs from all perspectives combined by over £204,704,386 and £307,056,580, respectively.

### Scenario analysis

**Fig. 3:**
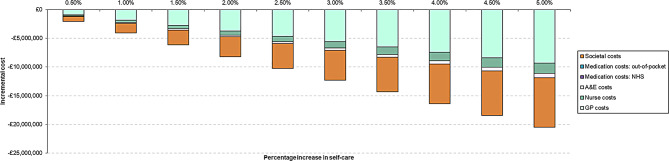
Incremental costs associated with various levels of increase in the use of self-care. A&E, Accident and emergency; GP, General practitioner; NHS, National Health Service

Figure 3 displays the cost-savings when the hypothetical increase in self-care ranges from 0.5% to 5%. Costs related to GP visits and absences from school and work contribute to the highest proportions of total cost savings (45% and 42%, respectively).

The results of the scenario analysis are presented in Supplementary Material S3. The societal cost savings associated with absenteeism increase from -£8,618,097 to -£9,475,754 when primary and secondary appointment waiting times are increased by 10%. The overall cost savings for all perspectives combined increase from -£20,470,439 to -£40,940,877 when the self-care uptake is increased to 10%. A hypothetical scenario in which it is not possible to purchase OTC ibuprofen indicates an increase of £910,925 and £15,324,153 in NHS and PSS, and out-of-pocket costs, respectively, when compared with current levels of self-care. The incremental costs associated with prescription-only ibuprofen are likely underestimated in this scenario because the number of healthcare appointments will increase. This scenario conservatively estimates the impact in terms of prescription costs only because it is not possible to determine the number of people who currently use self-care and, hence, would require an appointment to receive a prescription. Finally, the total opportunity cost-savings increased to £22,517,483 when the number of primary and secondary care appointments due to each condition was increased by 10%.

## Discussion

Previous research indicates that self-care may be underutilised within healthcare systems. For example, a discrete choice experiment conducted in the UK suggested that responders would favour self-care for minor conditions over pharmacy advice for primary care appointments: participants were willing to pay £22.62 for self-care in comparison to £19.13 or £17.18 for pharmacy advice or GP consultation, respectively [30]. Self-care is part of the NHS Long Term Plan, which introduces the concept of a more preventative healthcare model to improve NHS demand [3]. The Porteous et al. survey, carried out between April and June 2004, suggested that to gain the full benefits of such policies, the development of services must take people’s preferences into account [30].

The current study provides further evidence of the potential benefits of self-care, specifically related to opportunity cost savings to the healthcare system and wider societal gains from reduced absenteeism and presenteeism. Overall, the results indicate that an assumed increase in self-care could lead to a decrease in the number of primary and secondary care appointments, leading to opportunity cost savings for the NHS in the form of more appointments available for use by other people. The base case results found that a 5% increase in self-care with ibuprofen would result in opportunity cost savings of £11,852,342 from an NHS and PSS perspective across all three conditions, increasing to £20,470,439 when the societal perspective is considered.

DSA found the magnitude of change in self-care to be the main driver of the model results. The larger the increase in self-care, the greater the reduction in primary and secondary care appointment costs, and a greater reduction in waiting time and productivity loss. This was particularly true of the population with acute rhinosinusitis who experienced the greatest number of GP visits, and the population with migraine who experienced the greatest number of A&E visits. None of the variations examined in the DSA caused the direction of the model results to change.

The model only considered the cost implications of a change in self-care. Any clinical benefits, such as potential HRQoL improvements, were not captured. The impact of self-care on patient outcomes could be substantial and should be explored in the future. For example, self-care could be especially beneficial to the migraine population because earlier access to treatment can lead to shorter episodes of symptoms and higher pain-free rates compared with delayed treatment [3, 31]. Furthermore, only 77% of women in the UK feel comfortable discussing menstrual wellbeing with healthcare professionals [32]. If there was no access to self-care in the UK, a portion of the population may not feel comfortable accessing treatment for their symptoms through an NHS appointment. This could lead to a greater loss of earnings for a population with dysmenorrhoea as highlighted in a study by Ponzo et al. [33], which found that on average 5.8 days of work were missed due to the menstrual cycle in the last 12 months. A society without self-care would effectively be a less compassionate society. The Global Social and Economic Value of Self-Care 2022 report highlights the pillars of the social value of self-care which include quality of life, welfare and cost containment [4, 34]. The report states that an increase in self-care can lead to an increase in HRQoL because symptoms will be present for a shorter period and will have less intensity. In addition, self-care has been said to empower individuals to take responsibility for their healthcare and make healthier choices to live well [35].

A key strength of this analysis is that, to the authors’ knowledge, this is the first economic evaluation of self-care, specifically using ibuprofen to treat dysmenorrhoea, migraine and acute rhinosinusitis in the UK. Additionally, the inclusion of a societal perspective, which estimated both the number of school and work hours lost, as well as the cost associated with absenteeism, allowed for a more complete estimation of the overall impact of a change in self-care use. By estimating the out-of-pocket costs, the study highlights OTC ibuprofen as a cheaper alternative to prescription costs for individuals, as well as the opportunity costs to the NHS through issuing fewer prescriptions.

It was necessary to utilise several assumptions when completing the economic analysis. In particular, the potential magnitude of change in self-care used in the base case was hypothetical. Therefore, the number of primary and secondary care appointments that were estimated to be prevented in the model could be either an underestimate or an overestimate. However, it was not possible to determine the true potential change in self-care that would be feasible in NHS clinical practice, given the lack of data. The NHS already advocates for self-care as a first-line treatment, through initiatives such as National Self-Care Week, which promotes self-care across communities [36]. In addition, it also promotes a better use of the NHS by signposting people to the appropriate services based on their health needs. In particular, it provides information on when to refer to a pharmacist instead of a primary or secondary care professional in the first instance. Further research is required to determine how effective initiatives like these are and how much they influence self-care uptake to enable a realistic estimation.

It was also assumed that those using self-care would purchase medication immediately post-symptom onset, which would lead to the fast resolution of symptoms without the need for any primary or secondary care appointments. Therefore, no absence from work or school was necessary for these patients. It was conversely assumed that a proportion of those waiting for a healthcare appointment following symptom onset would not be well enough to attend school or work. It has also been implicitly assumed that these patients do not have any serious conditions that would have otherwise been diagnosed if they had attended an appointment. These are believed to be generalisable assumptions but will not reflect the experiences of all people with dysmenorrhoea, migraine and acute rhinosinusitis. The proportion of people required to take time off work for each condition was based on an assumption following a targeted literature review, which did not identify any relevant data. While the impact of education time lost could not be quantified, given the large heterogeneity associated with the impact on future productivity, published literature suggests that those who missed 5 days of school at 10 years of age are associated with 2% less earning at age 42 years, when compared with those who did not miss school. However, the authors report no significant differences in earnings after adjusting for confounders [37]. Sensitivity analysis was performed around these inputs and they did not cause an increase in self-care to become cost-increasing.

It was not possible to capture the true number of individuals who currently use self-care as a first-line treatment of minor conditions in the UK due to a paucity of data. Furthermore, the economic model only included three populations; those with dysmenorrhoea, migraine and acute rhinosinusitis. As such, this may have resulted in an underestimation of benefits, given that the current study has not included all conditions that could be treated using self-care instead of attending primary or secondary care appointments.

It was assumed that 60% of people receiving a prescription for dysmenorrhoea, migraine and acute rhinosinusitis are eligible for free prescriptions based on 40% of the UK population being liable to pay the prescription charge [38]. However, following clinical consultation and recent NHS policies on prescription medications for minor conditions, it was assumed that ibuprofen could not be acquired via a prescription and, therefore, all individuals in the model purchased OTC ibuprofen [2]. The out-of-pocket costs would be slightly lower for people who are eligible for free prescriptions and do not use self-care if ibuprofen could be acquired via prescription.

The model was populated with primary and secondary care data and costs from a UK perspective. However, the findings would likely be generalisable to other countries in which self-care can be used to treat any condition. For example, the impact of self-care has been explored in Malaysia, whereby several strategies including government regulation, increasing health literacy and engagement with communities and patient groups have been recommended [39]. Additionally, May and Bauer (2018) reported on the impact of self-care from a German healthcare system perspective [40]; the results support the outcomes of the current study, where it was concluded that each €1 spent by individuals on OTC medications leads to a €14 saved in the healthcare system and €4 saved on the national economy.

## Conclusions

An increase in self-care, when assumed to reduce unnecessary appointments, was shown to provide opportunity cost savings to the NHS, while also decreasing work and school absenteeism.

## Supplementary Information

Below is the link to the electronic supplementary material.

Supplementary Material 1

## Data Availability

All data generated or analysed during this study are included in this published article (and this supplementary material).

## Abbreviations

BNF: British National Formulary
DSA: Deterministic Sensitivity Analysis
GP: General Practitioner
NHS: National Health Service
NICE: National Institute for Health and Care Excellence
OTC: Over-the-counter
PSS: Personal Social Services
PSSRU: Personal Social Services Research Unit
UK: United Kingdom

## References

[1] British Medical Association. Self care: question & answer. 2019. Available from: https://www.bma.org.uk/media/1936/bma-plg-selfcare-nov-19.pdf

[2] NHS England. Policy guidance: conditions for which over the counter items should not be routinely prescribed in primary care. 2024. Available from: https://www.england.nhs.uk/long-read/policy-guidance-conditions-for-which-over-the-counter-items-should-not-be-routinely-prescribed-in-primary-care/

[3] Proprietary Association of Great Britain. A Self Care White Paper: supporting the delivery of the NHS Long Term Plan. 2019. Available from: https://www.pagb.co.uk/content/uploads/2019/03/PAGB_Self-Care_White-Paper_v1-0.pdf

[4] May U, Bauer C, Schneider-Ziebe A, Giulini-Limbach C. Self-care with non-prescription medicines to improve health care access and quality of life in low- and middle-income countries: systematic review and methodological approach. Front Public Health. 2023.11. 10.3389/fpubh.2023.1220984 PMC10525306 37771834

[5] Association of the European Self-Medication Industry. The Economic and Public Health Value of Self-Medication. Brussels:AESGP. 2004. Available from: https://aesgp.eu/content/uploads/2019/10/THE-ECONOMIC-AND-PUBLIC-HEALTH-VALUE-OF-SELF-MEDICATION.pdf

[6] Vowles K, Rosser B, Januszewicz P, Morlion B, Evers S, Eccleston C. Everyday pain, analgesic beliefs and analgesicbehaviours in Europe and russia: an epidemiologicalsurvey and analysis. Annual Hosp Pharm. 2017;1:40. 10.14748/.v1i1.1880.

[7] Armour M, Parry K, Manohar N, Holmes K, Ferfolja T, Curry C, et al. The prevalence and academic impact of dysmenorrhea in 21,573 young women: A systematic review and Meta-Analysis. J Womens Health (Larchmt). 2019;28(8):1161–71. 10.1089/jwh.2018.7615. 31170024 10.1089/jwh.2018.7615

[8] Jin X, Ren J, Li R, Gao Y, Zhang H, Li J, et al. Global burden of upper respiratory infections in 204 countries and territories, from 1990 to 2019. EClinicalMedicine. 2021;37:100986. 10.1016/j.eclinm.2021.100986. 34386754 10.1016/j.eclinm.2021.100986 PMC8343248

[9] National Institute for Health and Care Excellence (NICE). Scenario: Migraine in adults. 2022. Available from: https://cks.nice.org.uk/topics/migraine/management/adults/

[10] National Institute for Health and Care Excellence (NICE). Scenario: Migraine in young people aged 12–17 years. 2022. Available from: https://cks.nice.org.uk/topics/migraine/management/young-people-aged-12-17-years/

[11] National Institute for Health and Care Excellence (NICE). Scenario: Primary dysmenorrhoea. 2023. Available from: https://cks.nice.org.uk/topics/dysmenorrhoea/management/primary-dysmenorrhoea/

[12] National Institute for Health and Care Excellence (NICE). Scenario: Acute sinusitis. 2024.

[13] NHS Digital. Appointments in General Practice report. 2023. [cited September 2023 Available from: https://digital.nhs.uk/data-and-information/data-tools-and-services/data-services/general-practice-data-hub/appointments-in-general-practice

[14] NHS Digital. Hospital Accident & Emergency Activity 2021-22. 2022. Available from: https://digital.nhs.uk/data-and-information/publications/statistical/hospital-accident--emergency-activity/2021-22

[15] NHS. NHS prescription charges. 2023. [cited March 2024 Available from: https://www.nhs.uk/nhs-services/prescriptions/nhs-prescription-charges/

[16] Foden N, Burgess C, Shepherd K, Almeyda R. A guide to the management of acute rhinosinusitis in primary care management strategy based on best evidence and recent European guidelines. Br J Gen Pract. 2013;63(616):611–13. 10.3399/bjgp13X674620. 24267853 10.3399/bjgp13X674620 PMC3809423

[17] NHS England. News: Improved NHS migraine care to save thousands of hospital stays. 2020. Available from: https://www.england.nhs.uk/2020/01/improved-nhs-migraine-care/

[18] Jones K, Weatherly H, Birch S, Castelli A, Chalkley M, Dargan A et al. *Unit Costs of Health and Social Care 2022 Manual. 2023. Available from*: https://kar.kent.ac.uk/100519/.

[19] NHS England. 2021/22 National Cost Collection data. 2022. Available from: https://www.england.nhs.uk/costing-in-the-nhs/national-cost-collection/

[20] BMJ Best Practice. Assessment of dysmenorrhoea. 2024. [cited February 2024 Available from: https://bestpractice.bmj.com/topics/en-gb/420

[21] NHS. Health A to Z: Migraine. [cited September 2023 Available from: https://www.nhs.uk/conditions/migraine/

[22] Allan GM, Arroll B. Prevention and treatment of the common cold: making sense of the evidence. Can Med Assoc J. 2014;186(3):190–99. 10.1503/cmaj.121442. 24468694 10.1503/cmaj.121442 PMC3928210

[23] National Institute for Health and Care Excellence (NICE). Ibruprofen. 2024. Available from: https://bnf.nice.org.uk/drugs/ibuprofen/

[24] Boots. September. Nurofen 200 mg tablets – 12 tablets. [cited 2023 Available from: https://www.boots.com/nurofen-200mg-tablets-12-tablets-10067664

[25] Boots. Nurofen for Children 100 mg Chewable Capsules Orange – 12. [cited. September 2023 Available from: https://www.boots.com/nurofen-for-children-100mg-chewable-capsules-7-years-plus

[26] Boots. Nurofen for Children Strawberry 3 months to 12 years 100 mg/5 ml Oral Suspension 200 ml. [cited September 2023 Available from: https://www.boots.com/nurofen-for-children-strawberry-oral-suspension-200ml-10068984

[27] Office for National Statistics. National population projections: 2020-based interim, January 2022. 2022. Available from: https://www.ons.gov.uk/peoplepopulationandcommunity/populationandmigration/populationprojections/bulletins/nationalpopulationprojections/2020basedinterim

[28] Office for National Statistics. Annual Survey of Hours and Earnings time series of selected estimates. 2022. Available from: https://www.ons.gov.uk/employmentandlabourmarket/peopleinwork/earningsandworkinghours/datasets/ashe1997to2015selectedestimates

[29] Health Education and Improvement Wales (HEIW). NHS Wales Careers: General practice nurse. [cited. September 2023 Available from: https://heiw.nhs.wales/careers/nhs-wales-careers/roles/nursing/general-practice-nurse/

[30] Porteous T, Ryan M, Bond CM, Hannaford P. Preferences for self-care or professional advice for minor illness: a discrete choice experiment. Br J Gen Pract. 2006;56(533):911–7. 17132378 PMC1934050

[31] Scholpp J, Schellenberg R, Moeckesch B, Banik N. Early treatment of a migraine attack while pain is still mild increases the efficacy of Sumatriptan. Cephalalgia. 2004;24(11):925–33. 10.1111/j.1468-2982.2004.00802.x. 15482353 10.1111/j.1468-2982.2004.00802.x

[32] GOV.UK. Results of the. ‘Women’s Health – Let’s talk about it’ survey. 2022. Available from: https://www.gov.uk/government/calls-for-evidence/womens-health-strategy-call-for-evidence/outcome/results-of-the-womens-health-lets-talk-about-it-survey

[33] Ponzo S, Wickham A, Bamford R, Radovic T, Zhaunova L, Peven K, et al. Menstrual cycle-associated symptoms and workplace productivity in US employees: A cross-sectional survey of users of the Flo mobile phone app. Digit Health. 2022;8:20552076221145852. 10.1177/20552076221145852. 36544535 10.1177/20552076221145852 PMC9761221

[34] Global Self-Care Federation. The Global Social and Economic Value of Self-Care 2022. 2022. Available from: https://www.selfcarefederation.org/sites/default/files/media/documents/2022-06/FINAL_GSCF%20Socio-Economic%20Research%20Report%2022062022.pdf

[35] Proprietary Association of Great Britain (PAGB). A Self Care White Paper: supporting the delivery of the NHS Long Term Plan. 2019. Available from: https://www.pagb.co.uk/content/uploads/2019/03/PAGB_Self-Care_White-Paper_v1-0.pdf

[36] Forum S-CN, Self-Care W. 2024. Available from: https://www.selfcareforum.org/events/self-care-week/

[37] Dräger J, Klein M, Sosu E. The long-term consequences of early school absences for educational attainment and labour market outcomes. Br Edu Res J. 2024;n/a(n/a):1–19. 10.1002/berj.3992.

[38] GOV.UK. Free prescription age frozen at 60. 2023. [cited September 2023 Available from: https://www.gov.uk/government/news/free-prescription-age-frozen-at-60#:~:text=The%20current%20NHS%20prescription%20charge,are%20dispensed%20free%20of%20charge

[39] The Economist Intelligence Unit. Engage and educate: establishing self-care as a cornerstone to healthcare in Malaysia. 2020. Available from: https://www.phama.org.my/view_file.cfm?fileid=144

[40] May U, Bauer C. Phrmacy-based slef-care of minor ailments - a health economic analysis focused on the germ healthcare system. 2018. Available from: https://selfcarejournal.com/wp-content/uploads/2018/05/May-Bauer.-9.2.27-46.pdf

