# Supplementary material for: A health economics assessment of self-care with over-the-counter ibuprofen in dysmenorrhoea, migraine and acute rhinosinusitis in the United Kingdom
Source: Cost Eff Resour Alloc. 2025 Oct 15;23:56. doi: 10.1186/s12962-025-00660-6 (PMC12523155; doi:10.1186/s12962-025-00660-6)
Supplement: Supplementary file 1 — Supplementary Material 1 [file 12962_2025_660_MOESM1_ESM.docx]

**Supplementary material**

S1. Model Inputs

Table S. 1: Primary care visits per year

| **Number of visits per year under current practice** | **Dysmenorrhoea** | **Migraine** | **Acute rhinosinusitis** | **Source** |
| --- | --- | --- | --- | --- |
| Total number of appointments: GP | 158,566,452 | | | NHS Digital [1] |
| Total number of appointments: nurse | 71,731,639 | | |  |
| Proportion of primary care visits due to condition | 1.0% | 1.0% | 1.5% | Clinician consultation, NHS England [2]. Foden et al (2013) [3] |
| Proportion of appointments face-to-face (as opposed to via phone) | 70.8% | | | NHS Digital [1] |

GP, General practitioner; NHS, National Health Service.

**Table S. 2. Indirect costs and resource use**

| **Parameter** | **Value** | | | **Source** |
| --- | --- | --- | --- | --- |
| Average waiting time to see a GP or nurse following symptom onset | 1.28 days | | | NHS Digital [1] |
| Time spent in A&E | 3.90 hours | | | NHS Digital [4] |
| Average hourly pay | £14.79 | | | ONS [5] |
| Average working hours per week | 37.00 | | |  |
| Average school hours per week | 32.50 | | | Department for Education [6] |
| ***Population proportions*** | | | | |
| Proportion of the population aged 0 to 4 years old | 5.6% | | | ONS [7] |
| Proportion of the population aged 5 to 11 years old | 8.6% | | |  |
| Proportion of the population aged 12 to 17 years old | 6.9% | | |  |
| Proportion of the population aged 18 to 51 years old | 43.6% | | |  |
| Proportion of the population aged 52 to 64 years old | 16.6% | | |  |
| Proportion of the population aged 65 years and older | 18.6% | | |  |
| Proportion of dysmenorrhea patients (adults) | 86.4% | | | ONS [7] |
| Proportion of dysmenorrhea patients (children) | 13.6% | | |  |
| Proportion of population in school age (5-17 years old) | 15.5% | | |  |
| Proportion of population in working age (18-64 years old) | 60.2% | | |  |
| Proportion of population in retirement age (65 years or older) | 18.6% | | |  |
| **Parameter** | **Dysmenorrhoea** | **Migraine** | **Acute rhinosinusitis** | **Source** |
| Percentage of people who are off sick due to condition | 25% | 50% | 25% | Assumption |

A&E, Accident and emergency; GP, General practitioner; NHS, National Health Service; ONS, Office for National Statistics.

Table S. 3: Treatment costs by population

| **Parameter** | **Input** | | | **Source** |
| --- | --- | --- | --- | --- |
| Proportion of people under 12 years old | 14.3% | | | ONS [7] |
| Proportion of people 12 years or older | 85.7% | | |  |
| **Medication costs** | **% people** | **Unit cost** | **Mg per day** | **Source** |
| ***Generic drugs*** | | | | |
| Adults: ibuprofen 200mg tablets | 100% | £0.23 | 1,200 | Assumption, eMIT [8], NHS [9] |
| Children: ibuprofen: chewable capsule | 2% | £3.53 | 400 | Assumption, BNF [10], NHS [11] |
| Children: ibuprofen: oral suspension | 98% | £1.97 | 800 |  |
| ***Over-the-counter drugs*** | | | | |
| Adults: ibuprofen 200mg tablets | 100% | £1.99 | 1,200 | NHS [9], Boots [12] |
| Children: ibuprofen: chewable capsule | 2% | £5.45 | 400 | NHS [11], Boots [13] |
| Children: ibuprofen: oral suspension | 98% | £7.50 | 400 | NHS [11], Boots [14] |
| **Parameter** | **Dysmenorrhoea** | **Migraine** | **Acute rhinosinusitis** | **Source** |
| Average length of symptoms (days) | 3.00 | 1.54 | 2.00 | BMJ Best Practice [15], NHS [16], Allan, G.M & Arroll, B [17] |
| Weighted average drug cost per person per event (generic drugs) | £0.25 | £0.13 | £0.17 | NHS [9, 11], eMIT, [8], BNF [10] |
| Weighted average drug cost per person per event (over-the-counter drugs) | £2.99 | £1.35 | £1.76 | NHS [9, 11] , Boots [12-14] |

BNF, British National Formulary; eMIT, electronic market information tool; NHS, National Health Service.

**S2. Additional Base Case Results**

**Table S. 4. Cost breakdown by population**

|  | **Change in self-care** | **Current pathway** | **Difference** |
| --- | --- | --- | --- |
| **Dysmenorrhea** | | | |
| GP costs | £50,536,877 | £53,196,713 | -£2,659,836 |
| Nurse costs | £9,838,039 | £10,355,830 | -£517,792 |
| A&E costs | £1,422,380 | £1,497,243 | -£74,862 |
| Medication costs: NHS | £0 | £0 | £0 |
| Medication costs: out-of-pocket | £6,912,454 | £6,912,454 | £0 |
| Societal costs | £47,525,234 | £50,026,562 | -£2,501,328 |
| **Migraine** | | | |
| GP costs | £50,536,877 | £53,196,713 | -£2,659,836 |
| Nurse costs | £9,838,039 | £10,355,830 | -£517,792 |
| A&E costs | £8,711,229 | £9,169,715 | -£458,486 |
| Medication costs: NHS | £0 | £0 | £0 |
| Medication costs: out-of-pocket | £3,223,762 | £3,223,762 | £0 |
| Societal costs | £66,497,265 | £69,997,121 | -£3,499,856 |
| **Acute rhinosinusitis** | | | |
| GP costs | £75,805,316 | £79,795,069 | -£3,989,753 |
| Nurse costs | £14,757,058 | £15,533,745 | -£776,687 |
| A&E costs | £3,748,685 | £3,945,984 | -£197,299 |
| Medication costs: NHS | £0 | £0 | £0 |
| Medication costs: out-of-pocket | £6,133,199 | £6,133,199 | £0 |
| Societal costs | £49,721,336 | £52,338,248 | -£2,616,912 |

A&E, Accident and emergency; GP, General practitioner; NHS, National Health Service.

**S3. Scenario Analysis**

The scenarios described below were performed to access the robustness of the model results:

- Scenario 1: A scenario was performed to determine the impact on the costs associated with productivity loss if the waiting times for a healthcare appointment were increased by 10%.
- Scenario 2: A scenario was performed to estimate the possible cost savings if there was a 10% increase in the uptake of ibuprofen via self-care.
- Scenario 3: A scenario was performed in which a proportion of people would receive a prescription for ibuprofen during their appointment. Given that OTC ibuprofen is cheaper than a prescription, it was assumed that this option was only applicable to those eligible for free prescriptions (60% of the population) [18]. The cost of ibuprofen, when issued as a free prescription, was applied to the NHS and PSS perspective and was informed from NHS BNF and drugs and pharmaceutical electronic market information tool for the tablet and liquid formulations, respectively [8, 19] People who were not eligible for free prescriptions instead incurred an NHS prescription charge of £9.95 – these costs were applied to the out-of-pocket perspective [20]. Treatment costs for the proportion of people who were eligible for free prescriptions were applied to the NHS and PSS perspective.
- Scenario 4: A scenario was performed in which there was a 10% increase in the number of appointments for rhinosinusitis at baseline. This scenario was performed because the number of rhinosinusitis appointments is expected to be larger in winter.

Table S. 5. Summary of scenario analysis: all populations

|  | Change in self-care | Current pathway | Difference |
| --- | --- | --- | --- |
| **Scenario 1 – 10% increase in waiting time for primary and secondary care appointments** | | | |
| Total NHS and PSS costs | £225,194,499 | £237,046,841 | -£11,852,342 |
| Total out-of-pocket costs | £16,269,415 | £16,269,415 | £0 |
| Total societal costs | £180,039,329 | £189,515,084 | -£9,475,754 |
| Total costs | £421,503,244 | £442,831,340 | -£21,328,096 |
| **Scenario 2 - 10% increase in the uptake of self-care** | | | |
| Total NHS and PSS costs | £213,342,157 | £237,046,841 | -£23,704,684 |
| Total out-of-pocket costs | £16,269,415 | £16,269,415 | £0 |
| Total societal costs | £155,125,738 | £172,361,932 | -£17,236,193 |
| Total costs | £384,737,311 | £425,678,188 | -£40,940,877 |
| **Scenario 3 – ibuprofen not available over-the-counter** | | | |
| Total NHS and PSS costs | £237,957,767 | £237,046,841 | £910,925 |
| Total out-of-pocket costs | £31,593,568 | £16,269,415 | £15,324,153 |
| Total societal costs | £172,361,932 | £172,361,932 | £0 |
| Total costs | £441,913,266 | £425,678,188 | £16,235,079 |
| **Scenario 4 – 10% increase in the number of primary and secondary care appointments due to condition** | | | |
| Total NHS and PSS costs | £247,713,949 | £260,751,526 | -£13,037,576 |
| Total out-of-pocket costs | £17,896,356 | £17,896,356 | £0 |
| Total societal costs | £180,118,218 | £189,598,125 | -£9,479,906 |
| Total costs | £445,728,524 | £468,246,007 | -£22,517,483 |

NHS, National Health Service; PSS, Personal Social Services.

**Table S. 6. Summary of scenario analysis: dysmenorrhoea**

|  | **Change in self-care** | **Current pathway** | **Difference** |
| --- | --- | --- | --- |
| Base case | | | |
| Total NHS and PSS costs | £61,797,296 | £65,049,785 | -£3,252,489 |
| Total out-of-pocket costs | £6,912,454 | £6,912,454 | £0 |
| Total societal costs | £47,525,234 | £50,026,562 | -£2,501,328 |
| Total costs | £116,234,984 | £121,988,801 | -£5,753,817 |
| Work time lost | 3,721,117 hours | 3,916,966 hours | -195,848 hours |
| Education time lost | 446,024 hours | 469,499 hours | -23,475 hours |
| Total | 4,167,142 hours | 4,386,465 hours | -219,323 hours |
| Scenario 1 – 10% increase in waiting time for primary and secondary care appointments | | | |
| Total NHS and PSS costs | £61,797,296 | £65,049,785 | -£3,252,489 |
| Total out-of-pocket costs | £6,912,454 | £6,912,454 | £0 |
| Total societal costs | £52,254,823 | £55,005,077 | -£2,750,254 |
| Total costs | £120,964,573 | £126,967,316 | -£6,002,743 |
| Work time lost | 4,091,433 hours | 4,306,772 hours | -215,339 hours |
| Education time lost | 490,412 hours | 516,223 hours | -25,811 hours |
| Total | 4,581,845 hours | 4,822,995 hours | -241,150 hours |
| Scenario 2 – 10% increase in the uptake of self-care | | | |
| Total NHS and PSS costs | £58,544,807 | £65,049,785 | -£6,504,979 |
| Total out-of-pocket costs | £6,912,454 | £6,912,454 | £0 |
| Total societal costs | £45,023,906 | £50,026,562 | -£5,002,656 |
| Total costs | £110,481,166 | £121,988,801 | -£11,507,635 |
| Work time lost | 3,525,269 hours | 3,916,966 hours | -391,697 hours |
| Education time lost | 422,550 hours | 469,499 hours | -46,950 hours |
| Total | 3,947,819 hours | 4,386,465 hours | -438,647 hours |
| Scenario 3 – ibuprofen not available over-the-counter | | | |
| Total NHS and PSS costs | £65,403,862 | £65,049,785 | £354,076 |
| Total out-of-pocket costs | £8,938,717 | £6,912,454 | £2,026,264 |
| Total societal costs | £50,026,562 | £50,026,562 | £0 |
| Total costs | £124,369,141 | £121,988,801 | £2,380,340 |
| Work time lost | 3,916,966 hours | 3,916,966 hours | 0 hours |
| Education time lost | 469,499 hours | 469,499 hours | 0 hours |
| Total | 4,386,465 hours | 4,386,465 hours | 0 hours |
| Scenario 4 – 10% increase in the number of primary and secondary care appointments due to condition | | | |
| Total NHS and PSS costs | £67,977,026 | £71,554,764 | -£3,577,738 |
| Total out-of-pocket costs | £7,603,699 | £7,603,699 | £0 |
| Total societal costs | £52,277,757 | £55,029,218 | -£2,751,461 |
| Total costs | £127,858,482 | £134,187,681 | -£6,329,199 |
| Work time lost | 4,093,229 hours | 4,308,662 hours | -215,433 hours |
| Education time lost | 490,627 hours | 516,449 hours | -25,822 hours |
| Total | 4,583,856 hours | 4,825,112 hours | -241,256 hours |

NHS, National Health Service; PSS, Personal Social Services.

**Table S. 7. Summary of scenario analysis: migraine**

|  | **Change in self-care** | **Current pathway** | **Difference** |
| --- | --- | --- | --- |
| Base case | | | |
| Total NHS and PSS costs | £69,086,145 | £72,722,258 | -£3,636,113 |
| Total out-of-pocket costs | £3,223,762 | £3,223,762 | £0 |
| Total societal costs | £66,497,265 | £69,997,121 | -£3,499,856 |
| Total costs | £138,807,172 | £145,943,141 | -£7,135,969 |
| Work time lost | 7,468,875 hours | 7,861,974 hours | -393,099 hours |
| Education time lost | 1,017,979 hours | 1,071,557 hours | -53,578 hours |
| Total | 8,486,855 hours | 8,933,531 hours | -446,677 hours |
| Scenario 1 – 10% increase in waiting time for primary and secondary care appointments | | | |
| Total NHS and PSS costs | £69,086,145 | £72,722,258 | -£3,636,113 |
| Total out-of-pocket costs | £3,223,762 | £3,223,762 | £0 |
| Total societal costs | £73,115,017 | £76,963,176 | -£3,848,159 |
| Total costs | £145,424,924 | £152,909,196 | -£7,484,272 |
| Work time lost | 8,212,172 hours | 8,644,391 hours | -432,220 hours |
| Education time lost | 1,119,288 hours | 1,178,198 hours | -58,910 hours |
| Total | 9,331,460 hours | 9,822,589 hours | -491,129 hours |
| Scenario 2 – 10% increase in the uptake of self-care | | | |
| Total NHS and PSS costs | £65,450,032 | £72,722,258 | -£7,272,226 |
| Total out-of-pocket costs | £3,223,762 | £3,223,762 | £0 |
| Total societal costs | £62,997,409 | £69,997,121 | -£6,999,712 |
| Total costs | £131,671,203 | £145,943,141 | -£14,271,938 |
| Work time lost | 7,075,777 hours | 7,861,974 hours | -786,197 hours |
| Education time lost | 964,402 hours | 1,071,557 hours | -107,156 hours |
| Total | 8,040,178 hours | 8,933,531 hours | -893,353 hours |
| Scenario 3 – 0% of uptake in of self-care (everyone incurs NHS prescriptions charges) | | | |
| Total NHS and PSS costs | £72,914,110 | £72,722,258 | £191,852 |
| Total out-of-pocket costs | £9,190,895 | £3,223,762 | £5,967,133 |
| Total societal costs | £69,997,121 | £69,997,121 | £0 |
| Total costs | £152,102,126 | £145,943,141 | £6,158,985 |
| Work time lost | 7,861,974 hours | 7,861,974 hours | 0 hours |
| Education time lost | 1,071,557 hours | 1,071,557 hours | 0 hours |
| Total | 8,933,531 hours | 8,933,531 hours | 0 hours |
| Scenario 4 – 10% increase in the number of primary and secondary care appointments due to condition | | | |
| Total NHS and PSS costs | £75,994,760 | £79,994,484 | -£3,999,724 |
| Total out-of-pocket costs | £3,546,138 | £3,546,138 | £0 |
| Total societal costs | £73,146,992 | £76,996,834 | -£3,849,842 |
| Total costs | £152,687,889 | £160,537,455 | -£7,849,566 |
| Work time lost | 8,215,763 hours | 8,648,172 hours | -432,409 hours |
| Education time lost | 1,119,777 hours | 1,178,713 hours | -58,936 hours |
| Total | 9,335,540 hours | 9,826,885 hours | -491,344 hours |

NHS, National Health Service; PSS, Personal Social Services.

**Table S. 8. Summary of scenario analysis – acute rhinosinusitis**

|  | **Change in self-care** | **Current pathway** | **Difference** |
| --- | --- | --- | --- |
| Base case | | | |
| Total NHS and PSS costs | £94,311,058 | £99,274,798 | -£4,963,740 |
| Total out-of-pocket costs | £6,133,199 | £6,133,199 | £0 |
| Total societal costs | £49,721,336 | £52,338,248 | -£2,616,912 |
| Total costs | £150,165,593 | £157,746,246 | -£7,580,652 |
| Work time lost | 5,584,628 hours | 5,878,555 hours | -293,928 hours |
| Education time lost | 761,164 hours | 801,225 hours | -40,061 hours |
| Total | 6,345,791 hours | 6,679,780 hours | -333,989 hours |
| Scenario 1 – 10% increase in waiting time for primary and secondary care appointments | | | |
| Total NHS and PSS costs | £94,311,058 | £99,274,798 | -£4,963,740 |
| Total out-of-pocket costs | £6,133,199 | £6,133,199 | £0 |
| Total societal costs | £54,669,489 | £57,546,830 | -£2,877,342 |
| Total costs | £155,113,746 | £162,954,828 | -£7,841,081 |
| Work time lost | 6,140,397 hours | 6,463,576 hours | -323,179 hours |
| Education time lost | 836,913 hours | 880,961 hours | -44,048 hours |
| Total | 6,977,310 hours | 7,344,537 hours | -367,227 hours |
| Scenario 2 – 10% increase in the uptake of self-care | | | |
| Total NHS and PSS costs | £89,347,318 | £99,274,798 | -£9,927,480 |
| Total out-of-pocket costs | £6,133,199 | £6,133,199 | £0 |
| Total societal costs | £47,104,423 | £52,338,248 | -£5,233,825 |
| Total costs | £142,584,941 | £157,746,246 | -£15,161,305 |
| Work time lost | 5,290,700 hours | 5,878,555 hours | -587,856 hours |
| Education time lost | 721,102 hours | 801,225 hours | -80,122 hours |
| Total | 6,011,802 hours | 6,679,780 hours | -667,978 hours |
| Scenario 3 – 0% of uptake in self-care (everyone incurs NHS prescriptions charges) | | | |
| Total NHS and PSS costs | £99,639,796 | £99,274,798 | £364,997 |
| Total out-of-pocket costs | £13,463,955 | £6,133,199 | £7,330,756 |
| Total societal costs | £52,338,248 | £52,338,248 | £0 |
| Total costs | £165,441,999 | £157,746,246 | £7,695,754 |
| Work time lost | 5,878,555 hours | 5,878,555 hours | 0 hours |
| Education time lost | 801,225 hours | 801,225 hours | 0 hours |
| Total | 6,679,780 hours | 6,679,780 hours | 0 hours |
| Scenario 4 – 10% increase in the number of primary and secondary care appointments due to condition | | | |
| Total NHS and PSS costs | £103,742,164 | £109,202,278 | -£5,460,114 |
| Total out-of-pocket costs | £6,746,519 | £6,746,519 | £0 |
| Total societal costs | £54,693,469 | £57,572,073 | -£2,878,604 |
| Total costs | £165,182,153 | £173,520,870 | -£8,338,718 |
| Work time lost | 6,143,090 hours | 6,466,411 hours | -323,321 hours |
| Education time lost | 837,280 hours | 881,347 hours | -44,067 hours |
| Total | 6,980,370 hours | 7,347,758 hours | -367,388 hours |

NHS, National Health Service; PSS, Personal Social Services.

Figure S. 1: Tornado diagram: by population (all perspectives combined)

| **Panel a: Dysmenorrhoea** |
| --- |
| **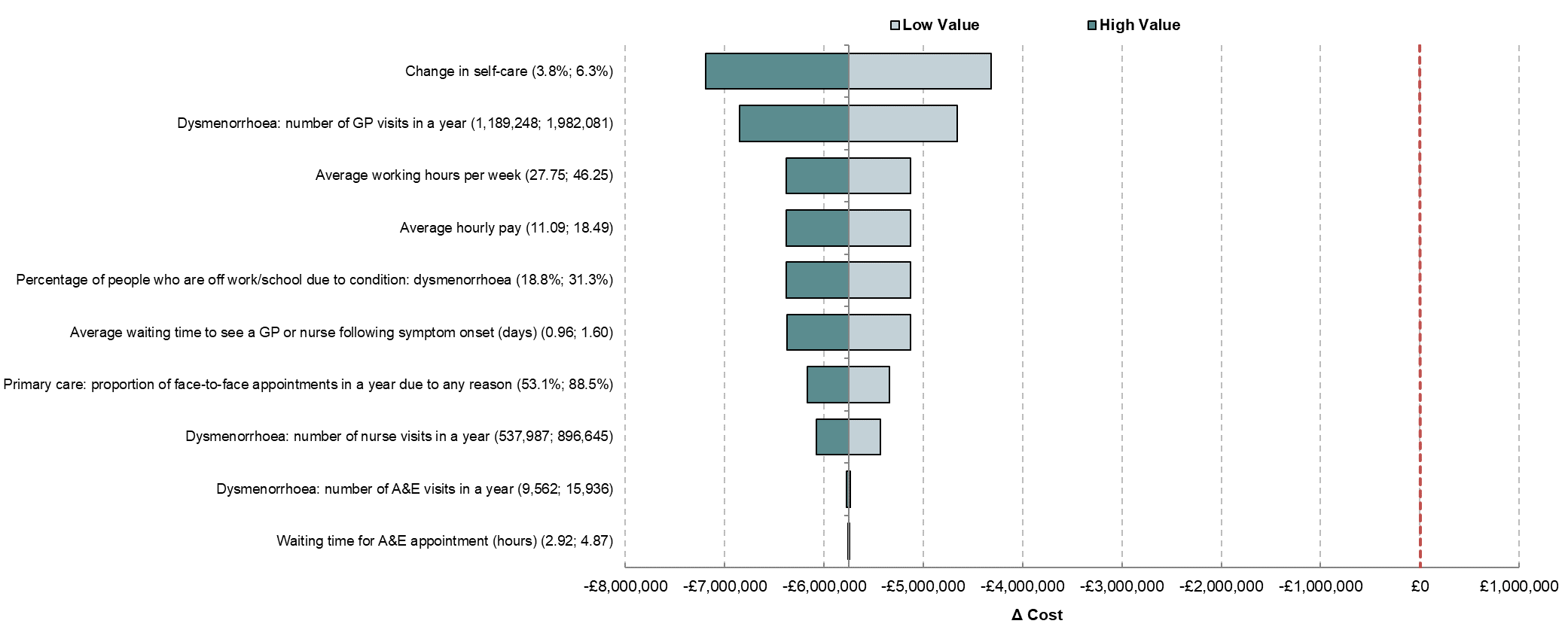** |
| **Panel b: Migraine** |
| **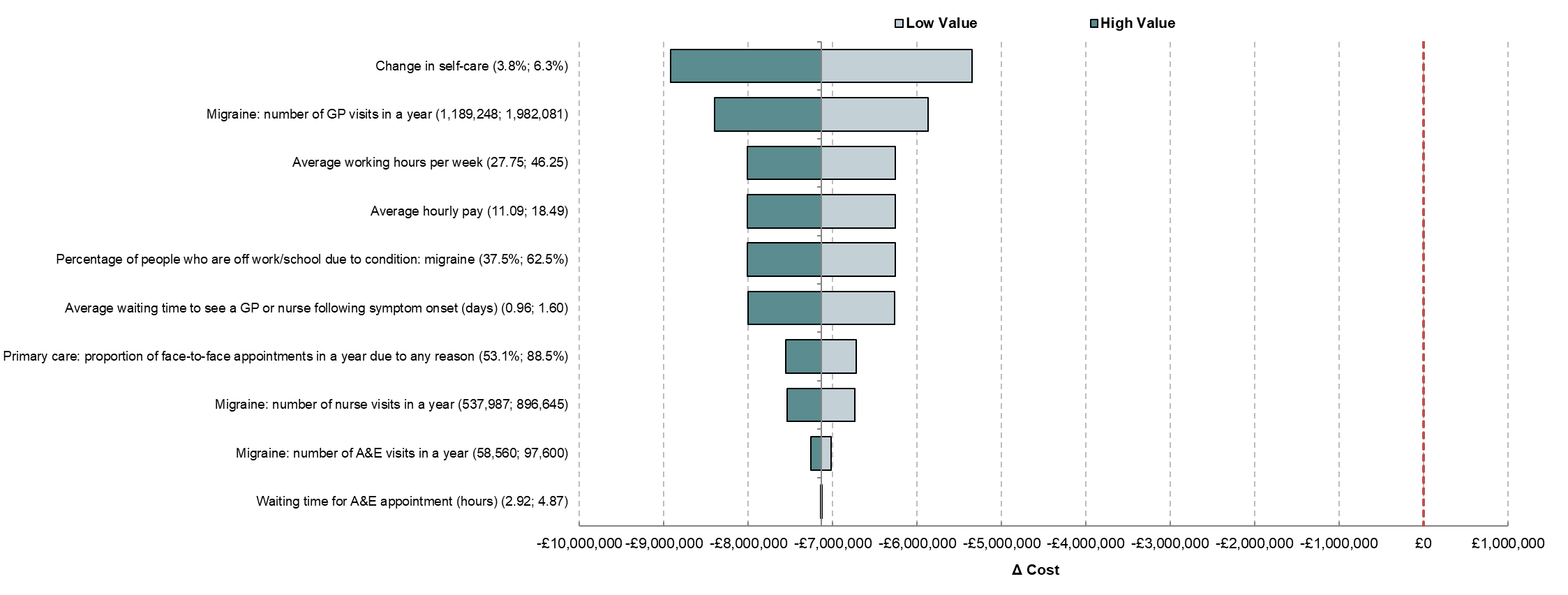** |
| **Panel c: Acute rhinosinusitis** |
| **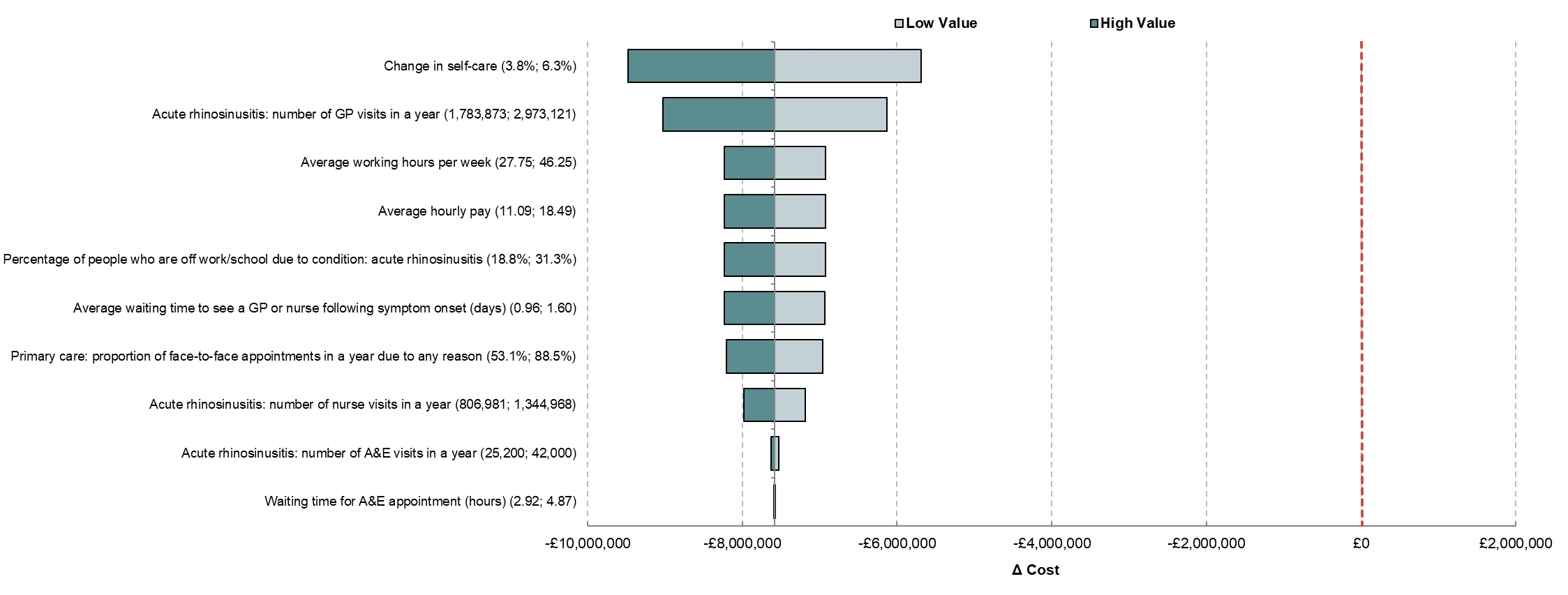** |

A&E, Accident and emergency; GP, General practitioner.

**Figure S. 2. Sensitivity analysis: incremental costs associated with various levels of decrease in the use of self-care (by population, all perspectives combined)**

**
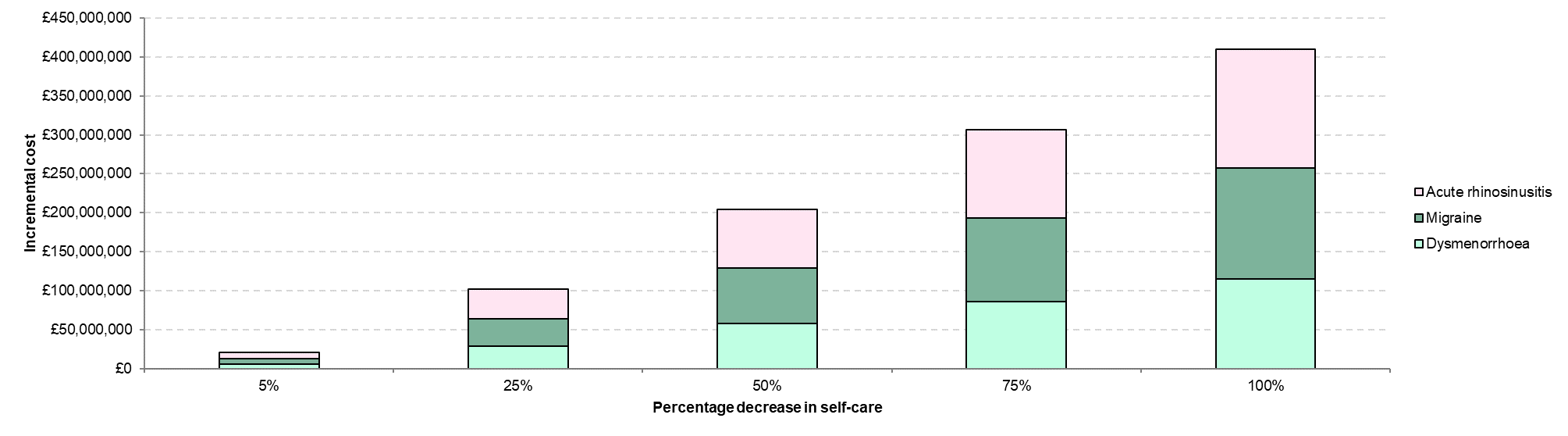
**

**References**

1. NHS Digital. Appointments in General Practice report. 2023. [cited September 2023 Available from: <https://digital.nhs.uk/data-and-information/data-tools-and-services/data-services/general-practice-data-hub/appointments-in-general-practice>.

2. NHS England. News: Improved NHS migraine care to save thousands of hospital stays. 2020. Available from: <https://www.england.nhs.uk/2020/01/improved-nhs-migraine-care/>.

3. Foden N, Burgess C, Shepherd K, Almeyda R. A guide to the management of acute rhinosinusitis in primary care management strategy based on best evidence and recent European guidelines. British Journal of General Practice. 2013;63(616):611-13.

4. NHS Digital. Hospital Accident & Emergency Activity 2021-22. 2022. Available from: <https://digital.nhs.uk/data-and-information/publications/statistical/hospital-accident--emergency-activity/2021-22>.

5. Office for National Statistics. Annual Survey of Hours and Earnings time series of selected estimates. 2022. Available from: <https://www.ons.gov.uk/employmentandlabourmarket/peopleinwork/earningsandworkinghours/datasets/ashe1997to2015selectedestimates>.

6. Department for Education. Length of the School Week non-statutory guidance. 2023. Available from: <https://assets.publishing.service.gov.uk/government/uploads/system/uploads/attachment_data/file/1167516/Minimum_School_Week_Non-Statutory_Guidance.pdf>.

7. Office for National Statistics. National population projections: 2020-based interim, January 2022. 2022. Available from: <https://www.ons.gov.uk/peoplepopulationandcommunity/populationandmigration/populationprojections/bulletins/nationalpopulationprojections/2020basedinterim>.

8. Department of Health and Social Care. Drugs and pharmaceutical electronic market information tool (eMIT). 2023. Available from: <https://www.gov.uk/government/publications/drugs-and-pharmaceutical-electronic-market-information-emit>.

9. NHS. Ibuprofen for adults (Nurofen). 2021. Available from: <https://www.nhs.uk/medicines/ibuprofen-for-adults/how-and-when-to-take-ibuprofen/>.

10. National Institute for Health and Care Excellence (NICE). British National Formulary (BNF): Ibuprofen. 2024. Available from: <https://bnf.nice.org.uk/drugs/ibuprofen/>.

11. NHS. Ibuprofen for children. 2022. Available from: <https://www.nhs.uk/medicines/ibuprofen-for-children/how-and-when-to-give-ibuprofen-for-children/>.

12. Boots. Nurofen 200mg tablets - 12 tablets. [cited September 2023 Available from: <https://www.boots.com/nurofen-200mg-tablets-12-tablets-10067664>.

13. Boots. Nurofen for Children 100mg Chewable Capsules Orange - 12. [cited September 2023 Available from: <https://www.boots.com/nurofen-for-children-100mg-chewable-capsules-7-years-plus>.

14. Boots. Nurofen for Children Strawberry 3 months to 12 years 100mg/5ml Oral Suspension 200ml. [cited September 2023 Available from: <https://www.boots.com/nurofen-for-children-strawberry-oral-suspension-200ml-10068984>

15. BMJ Best Practice. Assessment of dysmenorrhoea. 2024. [cited February 2024 Available from: <https://bestpractice.bmj.com/topics/en-gb/420>.

16. NHS. Health A to Z: Migraine. [cited September 2023 Available from: <https://www.nhs.uk/conditions/migraine/>.

17. Allan GM, Arroll B. Prevention and treatment of the common cold: making sense of the evidence. Canadian Medical Association Journal. 2014;186(3):190-99.

18. GOV.UK. Free prescription age frozen at 60. 2023. [cited September 2023 Available from: <https://www.gov.uk/government/news/free-prescription-age-frozen-at-60#:~:text=The%20current%20NHS%20prescription%20charge,are%20dispensed%20free%20of%20charge>.

19. National Institute for Health and Care Excellence (NICE). Ibruprofen. 2024. Available from: <https://bnf.nice.org.uk/drugs/ibuprofen/>.

20. NHS. NHS prescription charges. 2023. [cited March 2024 Available from: <https://www.nhs.uk/nhs-services/prescriptions/nhs-prescription-charges/>.
